# Can large language models help augment English psycholinguistic datasets?

**DOI:** 10.3758/s13428-024-02337-z

**Published:** 2024-01-23

**Authors:** Sean Trott

**Affiliations:** Department of Cognitive Science, UC San Diego, 9500 Gilman Dr., La Jolla, CA 92093-0515 USA

**Keywords:** Dataset, Psycholinguistic resource, Large language models, ChatGPT

## Abstract

Research on language and cognition relies extensively on psycholinguistic datasets or “norms”. These datasets contain judgments of lexical properties like concreteness and age of acquisition, and can be used to norm experimental stimuli, discover empirical relationships in the lexicon, and stress-test computational models. However, collecting human judgments at scale is both time-consuming and expensive. This issue of scale is compounded for multi-dimensional norms and those incorporating context. The current work asks whether large language models (LLMs) can be leveraged to *augment* the creation of large, psycholinguistic datasets in English. I use GPT-4 to collect multiple kinds of semantic judgments (e.g., word similarity, contextualized sensorimotor associations, iconicity) for English words and compare these judgments against the human “gold standard”. For each dataset, I find that GPT-4’s judgments are positively correlated with human judgments, in some cases rivaling or even exceeding the average inter-annotator agreement displayed by humans. I then identify several ways in which LLM-generated norms differ from human-generated norms systematically. I also perform several “substitution analyses”, which demonstrate that replacing human-generated norms with LLM-generated norms in a statistical model does not change the *sign* of parameter estimates (though in select cases, there are significant changes to their *magnitude*). I conclude by discussing the considerations and limitations associated with LLM-generated norms in general, including concerns of data contamination, the choice of LLM, external validity, construct validity, and data quality. Additionally, all of GPT-4’s judgments (over 30,000 in total) are made available online for further analysis.

**Supplementary Information:**

The online version contains supplementary material available at 10.3758/s13428-024-02337-z.

## Introduction

Research on language and cognition relies extensively on *large, psycholinguistic datasets*—sometimes called “norms”. These datasets contain information about various properties of words and sentences, including concreteness (Brysbaert et al., 2014a, b), sensorimotor associations (Lynott et al., 2020), affect (Bradley & Lang, 1999), semantic similarity (Hill et al., 2015), iconicity (Winter et al., 2023), and more.

Building these datasets is often time-consuming and expensive. One possible solution is to augment the construction of psycholinguistic datasets using computational tools, such as Large Language Models (LLMs), to reduce this difficulty; this approach is seeing growing popularity in related fields (Aher et al., 2022; Argyle et al., 2022; Törnberg, 2023; Zhu et al., 2023; Gilardi et al., 2023). However, the empirical question of whether and to what extent LLMs can reliably capture psycholinguistic judgments remains unanswered. In this paper, I apply LLMs to several major psycholinguistic datasets, quantify their performance, and discuss the advantages and disadvantages of this approach.

### Why do psycholinguists need psycholinguistic norms?

These norms have multiple uses. First, experimentalists can use this information to *normalize* (or “norm”) their stimuli. For example, a researcher designing a lexical decision task might ensure that the words in each condition are “matched” for properties such as frequency and concreteness to avoid introducing confounds.^1^

Second, these norms are often interesting to researchers in their own right. By examining the relationships between these properties, researchers can make inferences about the mechanisms that guide language acquisition, language processing and production, and even language change. For example, there is now a robust body of evidence (Thompson et al., 2012; Dingemanse et al., 2015; Winter et al., 2023) demonstrating a relationship between *iconicity* (the extent to which a word’s form resembles its meaning; e.g., the word “slurp” is often considered iconic) and *age of acquisition* (the average age at which a word is learned); this evidence—enabled by large-scale norms of iconicity and age of acquisition—informs theories of word learning (Dingemanse et al., 2015; Imai & Kita, 2014).

Importantly, while some of these norms can be estimated automatically from a large corpus of text (e.g., word frequency), many datasets rely on crowd-sourced human judgments. This raises a set of interrelated challenges for researchers interested in either creating or using these norms. In the section below, I describe why *scale* presents a central challenge, then introduce two other challenges—*multi-dimensional norms* and *context-dependent meaning*—that compound the problem of scale. I then introduce a potential solution—using large language models (LLMs) such as GPT-4 to augment the creation of psycholinguistic norms.

## The challenge of scale

It is both time-consuming and costly to collect these norms at *scale*. As a consequence, older datasets have been relatively small (e.g., fewer than 1000 words). Within recent decades, the development of online crowd-sourcing tools like Amazon Mechanical Turk^2^ has enabled larger-scale datasets containing judgments for thousands of words (Winter et al., 2023; Brysbaert et al., 2014a, b).

However, creating these datasets remains a non-trivial task: if the goal is to collect ten judgments each for 40,000 words, a researcher must collect a total of 400,000 judgments. Assuming each judgment takes approximately 5~s to make, then even a researcher paying the federal minimum wage ($7.25 per hour^3^) will need to pay at least $4000. And as others have noted (Webb & Tangney, 2022; Veselovsky et al., 2023), many participants on websites like Amazon Mechanical Turk are unreliable, which necessitates the use of rigorous exclusion criteria. Assuming a 25% exclusion rate (substantially lower than the rate reported by Webb & Tangney, 2022), a researcher would need to collect approximately 533,333 judgments, for a total cost of at least $5413.

This problem of scale is compounded by two sub-problems: the desire to collect *multi-dimensional norms* for words; and second, the fact that certain semantic properties vary across *contexts*.

- **Sub-problem #1: Multi-dimensional norms.** Researchers are increasingly interested in *multi-dimensional properties* of words. For example, the Lancaster sensorimotor norms contain judgments about the extent to which six sensory modalities and five action effectors are associated with different words (Lynott et al., 2020); these norms, along with others (Binder et al., 2016), offer a degree of granularity and semantic nuance that is much harder to achieve with a single dimension. Yet as more dimensions of interest are added, the problem of scale compounds. Returning to the example above: if a researcher needs ten judgments for *11 different dimensions* of 40,000 words, which amounts to 4,400,00 judgments in total.
- **Sub-problem #2: Context.** Words mean different things in different contexts (Yee & Thompson-Schill, 2016). This is most obvious in cases of lexical ambiguity (e.g., “wooden *table*” vs. “data *table*”), but it arguably also applies to more subtle contextual variation (e.g., “She cut the *lemon*” vs. “She juggled the *lemon*”). This poses a potential problem for norms of words judged in isolation. For example, the word “table” would likely be judged as more concrete in “wooden *table*” than “data *table*”; similarly, the word “market” has more olfactory associations in the expression “fish *market*” than “stock *market*” (Trott & Bergen, 2022).

There have been some efforts to collect semantic norms in context (Scott et al., 2019; Trott & Bergen, 2021; Haber & Poesio, 2021; Trott & Bergen, 2022). Here, however, scale is even more challenging, as the number of contexts in which a word might appear is potentially infinite. Which contexts should a researcher collect judgments for?

This also inherently limits the utility of these datasets for researchers wishing to norm stimuli that consist of more than single words in isolation. For example, perhaps a researcher wishes to measure behavioral or neurophysiological responses to ambiguous words in different contexts (e.g., “wooden *table*" vs. “data *table*”). In this case, the researcher may wish to norm their stimuli not only for the concreteness of the target word in isolation (e.g., “table”), but also for concreteness of that particular meaning in a given context (e.g., “wooden *table*”). Yet it is very unlikely that contextualized norms for these exact sentences would already exist, precisely because there are an infinite number of contexts in which a given word could occur—thus requiring researchers to collect their own norms. This means that even a very large dataset of contextualized judgments may have limited direct practical application.

### Can LLMs helps scale psycholinguistic norms?

The current work investigates a potential solution to this problem: using large language models (LLMs) to augment psycholinguistic datasets. If LLMs provide judgments that are sufficiently humanlike, then a small dataset of human norms (i.e., a “gold standard”) could be rapidly *scaled* to encompass more words, more semantic dimensions, and more contexts—all at substantially lower cost. If, however, LLMs prove unreliable—i.e., their responses diverge too much from those of humans—then it is important to quantify both the *source* and *extent* of this unreliability. This question is especially pressing because there is a growing body of research interested in using LLMs as experimental subjects and data annotators (Aher et al., 2022; Argyle et al., 2022; Törnberg, 2023; Zhu et al., 2023; Gilardi et al., 2023; Jain et al., 2023).

#### Related work

Scaling psycholinguistic datasets with the aid of computational techniques has been a longstanding goal of both psycholinguistics and natural language processing. The majority of these approaches rely on some level on the *distributional hypothesis*, namely that “you shall know a word by the company it keeps” (Firth, 1957, pg. 11; Lewis et al., 2019). If words with similar meanings appear in similar contexts (Harris, 1954; McDonald & Ramscar, 2001), then the meaning of a word can be inferred in part by observing the contexts in which it occurs.

While early work (Hatzivassiloglou & McKeown, 1997) relied primarily on count-based methods, the development of more advanced computational techniques and accessibility to larger corpora gave researchers additional power and flexibility. Algorithms like *word2vec* (Mikolov et al., 2013) allow researchers to represent words as *vectors* (or “word embeddings”) of real numbers, which can in turn be used in various arithmetic and statistical operations. Most relevantly, these representations can be leveraged to train a statistical model to predict psycholinguistic dimensions of interest, such as concreteness or arousal (Bestgen & Vincze, 2012). These representations have been leveraged to extend norms across languages (Thompson & Lupyan, 2018) and in some cases (Utsumi, 2020), to norms with many semantic dimensions (Binder et al., 2016).

Notably, however, the approaches reviewed above all focus on predicting semantic properties of *words in isolation*; this is in large part because of limitations in computational techniques at the time the studies were conducted.

#### Large language models: Advances and breakthroughs

Recent advances—including access to more training data, more sophisticated neural network architectures, and more computing resources—have led to remarkable improvements in language models (Ouyang et al., 2022; Katz et al., 2023). Because of their size (often billions of parameters), these systems are sometimes called large language models (LLMs). Contemporary LLMs are artificial neural networks, which learn to map some *input* representation to an *output* by iteratively tuning the *weights* between neurons in different layers of the network. This tuning process is achieved by extensive training. Specifically, LLMs are trained using a *token prediction* paradigm: given a string of tokens (e.g., “The cat sat on the ___”), an LLM must predict which word is most likely to come next.^4^By observing many sentences like this one, an LLM eventually tunes its weights to produce more accurate probability distributions over the upcoming token (e.g., “mat”).

Importantly, LLMs trained in this way display behavior consistent with the acquisition of both syntactic and semantic knowledge (Tenney et al., 2019). Metrics derived from LLMs also successfully predict *human* dependent measures on a number of psycholinguistic tasks (Michaelov et al., 2022; Shain, 2019), and are sensitive to relevant psycholinguistic constructs (Trott & Bergen, 2023; Li & Joanisse, 2021; Jones et al., 2022; Trott et al., 2022). Critically for our purposes, LLMs are sensitive to *context*: that is, an LLM’s representation of a given word (e.g., “bank”) differs on the basis of the immediate linguistic context for that word (e.g., “financial bank” vs. “river bank”). This makes LLMs potentially well suited for research questions that ask how context modifies behavioral responses to a given stimulus or prompt.

#### LLMs as experimental subjects and data annotators

Improvements in LLMs have in turn led to an explosion of interest in using LLMs in behavioral research, either to replace crowd-sourced workers (Törnberg, 2023; Zhu et al., 2023; Gilardi et al., 2023) or even as experimental subjects (Aher et al., 2022; Argyle et al., 2022; Dillion et al., 2023; Jain et al., 2023; Cai et al., 2023; Binz & Schulz, 2023; Coda-Forno et al., 2023; Hagendorff, 2023; Kosinski, 2023). In the former case, some efforts have focused on tagging social media data (Törnberg, 2023; Zhu et al., 2023; Gilardi et al., 2023) with relevant information such as sentiment and the topic being discussed. Other researchers have explored the use of LLMs in fact-checking (Hoes et al., 2023) and analyzing text for offensiveness or sentiment (Rathje et al., 2023), albeit with mixed results (Ollion et al., 2023). In the latter case, LLMs have been used as subjects for a diverse array of experimental tasks, involving decision-making (Coda-Forno et al., 2023), Theory of Mind (Trott et al., 2022; Kosinski, 2023), sound symbolism (Cai et al., 2023), moral evaluation (Dillion et al., 2023), and more. As others have noted (Jain et al., 2023; Doerig et al., 2023), one benefit of LLMs is that they allow for *in silico* experimentation, allowing researchers to rapidly develop and test novel hypotheses.

This surge of interest is exciting; advances in LLMs represent a genuine opportunity for the field of cognitive science. One of those opportunities could be helping scale psycholinguistic datasets. Yet to my knowledge, this question has not been investigated in a systematic way. As behavioral researchers, an empirical, rigorous evaluation of how well state-of-the-art LLMs estimate psycholinguistic norms is critical for making informed decisions about whether and to what extent to incorporate LLMs into our research agenda. If LLMs perform poorly, it is premature to even consider incorporating them; if they perform well, this could pave the way for a more thorough research program on whether LLMs might serve a useful purpose. Such a research program could investigate not only how LLMs behave but also the internal representations that guide that behavior (Pavlick, 2023). However, establishing their performance empirically is a crucial first step in either direction.

## Current work

The primary goal of the current work was to investigate the viability of using state-of-the-art LLMs to augment psycholinguistic norms. I selected a state-of-the-art LLM (GPT-4) and elicited judgments for a number of psycholinguistic norms; I then quantified the extent to which LLM-generated norms correlated with those produced by humans. Datasets with *contextualized judgments* (Trott & Bergen, 2022; Scott et al., 2019; Trott & Bergen, 2020) were prioritized: as mentioned in the Introduction, these are intrinsically challenging to scale with human participants, so establishing the viability of LLM-generated judgments was of particular interest. Additionally, I selected several datasets containing judgment types that were either known to be challenging for language models, e.g., similarity judgments (Hill et al., 2015), or seemed a priori like psycholinguistic dimensions that should be challenging for an LLM, e.g., iconicity (Winter et al., 2023). The primary analyses for four of the six datasets considered were pre-registered on the Open Science Framework (individual links can be found in the Methods section). Additionally, the LLM-generated norms, along with the code required to reproduce the analyses described below, can all be found on GitHub (https://github.com/seantrott/llm_norms).

### Methods

#### Datasets

Six datasets were considered. Three of these datasets involved contextualized judgments. First, the Glasgow Norms (Scott et al., 2019) contain judgments about nine semantic dimensions (concreteness, age of acquisition, semantic size, valence, arousal, semantic gender, semantic dominance, familiarity, and imageability) for English words; I selected the subset of the norms containing contextualized judgments for 379 ambiguous words, e.g., “bow (ribbon)” vs. “bow (ship)”. Second, the Contextualized Sensorimotor Norms (Trott & Bergen, 2022) contain judgments about the relative strength for six sensory modalities (e.g., vision, hearing, etc.) and five action effectors (e.g., Hand/Arm, Foot/Leg, etc.) for 112 English words, in four different sentential contexts each (for a total of 448 sentences). Third, the RAW-C dataset (Relatedness of Ambiguous Words—in Context) contains judgments about the *relatedness* between the same ambiguous English word in distinct sentential contexts (e.g., “She liked the marinated lamb” vs. “She liked the friendly lamb”); RAW-C contains a total of 672 sentence pairs (Trott & Bergen, 2021).

I also considered three datasets involving judgments of individual words. SimLex999 (Hill et al., 2015) and SimVerb3500 (Gerz et al., 2016) contain judgments of *similarity* (as opposed to relatedness) of 999 word pairs and 3500 verb pairs, respectively. Finally, a recent dataset of *iconicity* judgments (the extent to which a word’s form resembles its meaning) for 14,776 English words was included (Winter et al., 2023).

#### Model

I used GPT-4, a state-of-the-art large language model. The primary reason for selecting GPT-4 was its superior performance on a number of natural language processing benchmarks, as well as more general metrics of capability.^5^

There are two limitations to using GPT-4 as a model: first, the details of model’s architecture and data are still unclear; and second, output can be obtained only by generating tokens (see “Prompting”), as opposed to accessing individual log probabilities—which may underestimate its performance (Hu & Levy, 2023). In my view, the superior performance of GPT-4 relative to competitors outweighed its downsides, particularly because the emphasis of the current work is on establishing the viability of the method, as opposed to probing the internal mechanics of the model itself.

#### Prompting

One benefit of modern LLMs is that they can be “prompted” using approaches not unlike giving instructions to human participants. I accessed GPT-4 using the OpenAI Chat Completion API.^6^ For each item in each dataset, GPT-4 was presented with instructions that matched the original instructions given to human participants as closely as possible. The temperature was set to 0, and GPT-4 was allowed to generate up to ten tokens in response.

In the case of the Glasgow Norms (Scott et al., 2019), the instructions were modified slightly to specify that GPT-4 should respond with a number between 1 and 7 (except for Arousal, Valence, and Dominance, for which the scales ranged from 1 to 9).^7^ Additionally, for Age of Acquisition, the pre-registered prompt asked GPT-4 to respond with the *age* at which a word was learned, whereas the original Glasgow Norms map ages to a 1–7 scale; I converted GPT-4’s raw age responses to a 1–7 scale using the mapping provided in the paper’s supplementary materials (Scott et al., 2019).^8^

For all datasets, the instructions, along with the item in question, were presented in entirety as a string input to GPT-4; this string included a line-separated prompt for GPT-4 to indicate its answer (e.g., “Iconicity: ”, or “Relatedness: ”). For datasets involving multiple semantic dimensions each item, the item was presented multiple times to GPT-4 (as independent “trials”) with modified instructions (e.g., according to the semantic dimension in question). This approach was chosen to avoid confounding responses between dimensions or between items.

The prompting method (and primary analyses) were pre-registered for: the iconicity norms (https://osf.io/wn9pv), SimVerb3500 (https://osf.io/dtekj), the contextualized sensorimotor norms (https://osf.io/2e3vk), and the Glasgow Norms (https://osf.io/3jvg6).

#### Processing

The output of the prompting procedure (a *.txt* file) was converted to a *.csv* file with the appropriate column headers (e.g., “Word”, “Sentence”, “Visual Strength”). Additionally, GPT-4’s response (originally a string, e.g., “1”) was converted to a number (e.g., 1). In three cases, no number could be identified in GPT-4’s response: in each case, GPT-4’s response indicated a refusal to answer the question. As decided in the pre-registration, those responses were excluded.

### Results

#### Primary analysis: Assessing GPT-4’s performance

The primary question was whether GPT-4’s ratings significantly co-varied with human ratings. To measure degree-of-fit, I calculated the Spearman’s rank correlation coefficient between GPT-4’s ratings and the human ratings; for datasets containing multiple dimensions (Trott & Bergen, 2022; Scott et al., 2019), I calculated *rho* for each dimension. In all cases, *rho* was positive and significantly above zero (*p* < .001), demonstrating that GPT-4’s rating’s captured relevant variance about the human ratings. Degree-of-fit ranged from a low of 0.39 (for semantic dominance) to a high of 0.86 (for similarity). For contextualized datasets, the highest *rho* achieved was 0.82 (for contextualized relatedness). The full set of correlation coefficients can be found in Table 1.

**Table 1:** Spearman’s rank correlation coefficients for each semantic dimension of each dataset

Dataset	Dimension	Contextualized?	Spearman’s *rho*
Iconicity norms (Winter et al., 2023)	Iconicity	No	0.59
SimLex999 (Hill et al., 2015)	Similarity	No	0.86
SimVerb3500 (Gerz et al., 2016	Similarity	No	0.81
RAW-C (Trott & Bergen, 2021)	Relatedness	Yes	0.82
CS Norms: Perception (Trott & Bergen, 2022)	All perception dimensions	Yes	0.84
CS Norms: Perception (Trott & Bergen, 2022)	Interoception	Yes	0.55
CS Norms: Perception (Trott & Bergen, 2022)	Taste	Yes	0.63
CS Norms: Perception (Trott & Bergen, 2022)	Hearing	Yes	0.66
CS Norms: Perception (Trott & Bergen, 2022)	Vision	Yes	0.66
CS Norms: Perception (Trott & Bergen, 2022)	Olfaction	Yes	0.71
CS Norms: Perception (Trott & Bergen, 2022)	Touch	Yes	0.75
CS Norms: Action (Trott & Bergen, 2022)	All action dimensions	Yes	0.64
CS Norms: Action (Trott & Bergen, 2022)	Head	Yes	0.45
CS Norms: Action (Trott & Bergen, 2022)	Mouth/Throat	Yes	0.56
CS Norms: Action (Trott & Bergen, 2022)	Foot/Leg	Yes	0.56
CS Norms: Action (Trott & Bergen, 2022)	Torso	Yes	0.58
CS Norms: Action (Trott & Bergen, 2022)	Hand/Arm	Yes	0.64
Glasgow Norms (Scott et al., 2019)	Valence	Yes	0.76
Glasgow Norms (Scott et al., 2019)	Arousal	Yes	0.66
Glasgow Norms (Scott et al., 2019)	Concreteness	Yes	0.81
Glasgow Norms (Scott et al., 2019)	Familiarity	Yes	0.71
Glasgow Norms (Scott et al., 2019)	Imageability	Yes	0.74
Glasgow Norms (Scott et al., 2019)	Dominance	Yes	0.39
Glasgow Norms (Scott et al., 2019)	AoA	Yes	0.72
Glasgow Norms (Scott et al., 2019)	Size	Yes	0.69
Glasgow Norms (Scott et al., 2019)	Gender	Yes	0.47

Another key question was how GPT-4’s performance compared to *human inter-annotator agreement* for that dataset. This is important as a baseline: if human agreement is low, then it is unreasonable to expect GPT-4’s performance to be very high. Here, the most comparable measure was leave-one-out inter-annotator agreement,^9^ which calculates the correlation between each human’s ratings and the mean ratings of all other human participants (excluding the participant in question). This information was available only for select datasets (Trott & Bergen, 2021; Trott & Bergen, 2022; Hill et al., 2015; Gerz et al., 2016; Winter et al., 2023); additionally, for the contextualized sensorimotor norms (Trott & Bergen, 2022), the published inter-annotator agreement measure was calculated aggregating across the entire set of action norms (five in total) and perception norms (six in total) but not each individual dimension. For the iconicity norms (Winter et al., 2023), I calculated leave-one-out inter-annotator agreement using the full raw data publicly available online, after applying the exclusion criteria that were possible given the data contents (i.e., all but the attention checks).

**Fig. 1:**
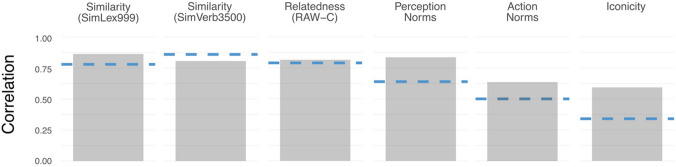
Spearman’s rho between GPT-4’s ratings and the human ratings for datasets with human inter-annotator agreement available. Human inter-annotator agreement is visualized in *blue*. For each dataset but SimVerb3500, GPT-4’s ratings were at least as correlated with the gold standard as the average inter-annotator agreement

Figure 1 below thus compares *rho* for GPT-4 to the average inter-annotator agreement among datasets for which it was available. Notably, in all but one dataset (SimVerb3500), GPT-4’s correlation with human ratings was *at least as high* as average inter-annotator agreement. Put another way: GPT-4 was more correlated with the average human rating (the population parameter) than the average human was. (Explanations for this phenomenon will be explored in the General discussion.)

Another way to assess the validity of these ratings is to ask about their relationship to *independently* collected judgments for the same measure. For example, Winter et al. (2023) compared their iconicity ratings to previously published judgments for a subset of the same words (Perlman et al., 2015), and obtained Pearson’s correlation coefficients between 0.48 (for auditory stimuli) and 0.55 (for written stimuli). By comparison, the Pearson’s correlation between GPT-4’s ratings and the Winter et al. (2023) ratings was *r* = 0.63. GPT-4’s ratings were also *more* correlated with the Perlman et al. (2015) ratings, for both auditory stimuli (*r = 0.53, p <* .001) and written stimuli (*r =* 0.58,* p* < .001). This is further evidence for the reliability of the GPT-4 ratings. (Additionally, data contamination from previously published datasets is unlikely to be the explanation here: see Supplementary Analysis 1 for more details.)

**Fig. 2:**
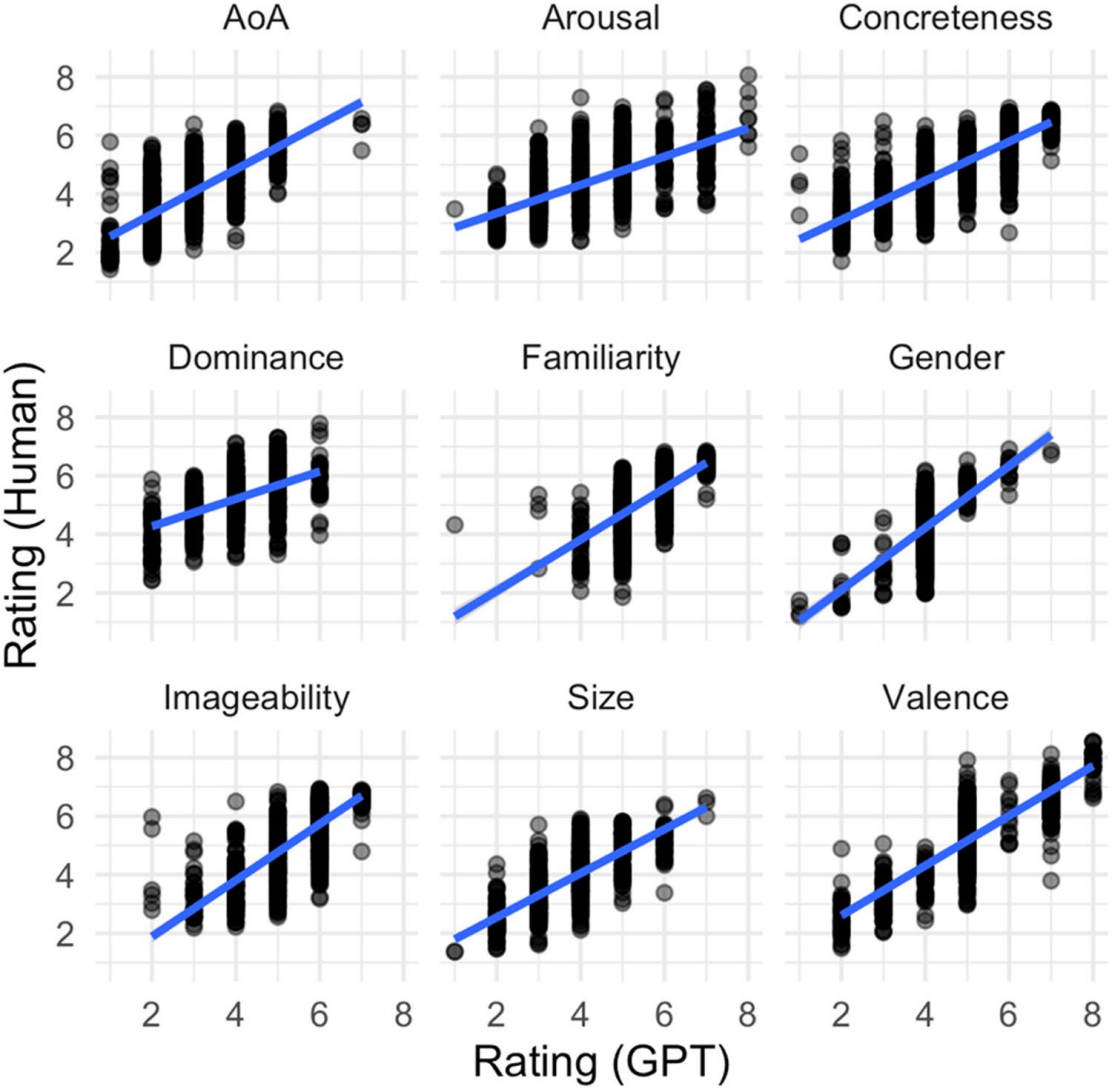
Relationship between GPT-4’s ratings and human ratings for each dimension of the Glasgow Norms. Note that the rating scale ranged between 1 and 7 for six of the nine norms, and between 1 and 9 for Arousal, Valence, and Dominance. Highest performance was achieved for Concreteness (*rho* = 0.81), and the lowest correlation was for dominance (*rho* = 0.39). GPT-4 ratings were significantly and positively correlated with human ratings for all dimensions

Focusing specifically on the Glasgow Norms (Scott et al., 2019), GPT-4’s performance ranged considerably, from relatively low correlations for contextualized semantic dominance (*rho = 0.39*) to very high correlation for contextualized concreteness (*rho = 0.81*). The relationship between GPT-4’s ratings and human ratings for each dimension of the Glasgow Norms is depicted in Fig. 2. Because inter-annotator agreement ratings were not available for the Glasgow Norms, it is more challenging to assess whether this range mirrors agreement observed for humans.

#### Does GPT-4 make systematic errors?

GPT-4’s ratings correlate with human ratings, but not perfectly. Is it possible to identify *systematic* sources of divergence, or are GPT-4’s errors randomly distributed? I attempted to address this question using available covariates for SimLex999, SimVerb3500, and RAW-C. These analyses were motivated by past work (Utsumi, 2020; Dou et al., 2018; Trott & Bergen, 2023), but also exploratory in nature. In each case, I quantified the absolute error between GPT-4’s ratings and the human ratings.

**Fig. 3:**
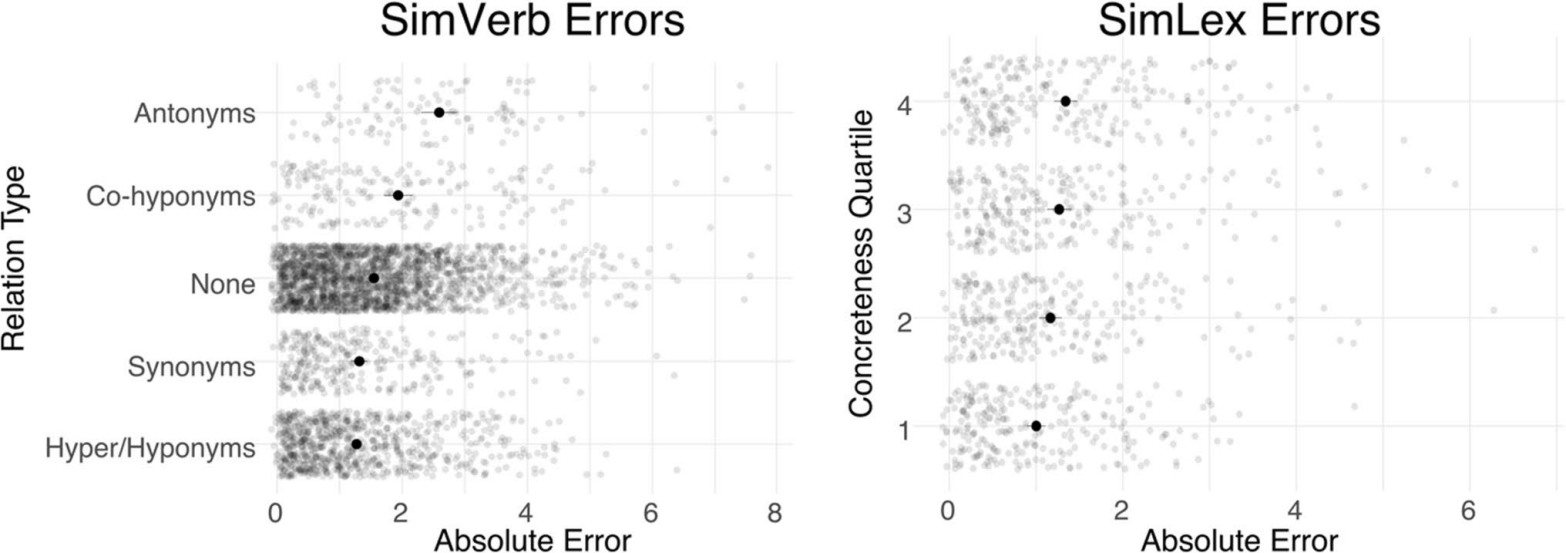
Absolute differences between GPT-4’s similarity ratings and human similarity ratings for SimVerb3500 and SimLex999. For SimVerb3500, errors were largest for Antonyms; for SimLex999, errors were larger for more concrete word pairs than more abstract word pairs

*SimLex999.* The SimLex999 dataset (Hill et al., 2015) contains information about both the *part-of-speech* of the two words being compared as well as their *concreteness* (binned by quartile). A linear regression predicting absolute error, with both factors as predictors, suggested independent effect of each: word pairs in higher concrete quartiles were associated with higher error [*β* = 0.15, SE = 0.04,* p* < .001]; additionally, verbs were also associated with higher error than adjectives [*β* = 0.55, SE = 0.12*, p <* .001]. The former effect is consistent with convergent evidence that distributional information is better at predicting semantic properties of abstract words than concrete words (Utsumi, 2020; Kiros et al., 2018). These effects, along with the analysis of errors for SimVerb3500, are displayed in Fig. 3. Qualitative inspection revealed that some of the highest divergences were for word pairs that formed semantic complements in some way, e.g., “wife/husband”, “south/north”, and “groom/bride”.

*SimVerb3500*. The SimVerb3500 dataset (Gerz et al., 2016) is annotated for the type of *relation* between the verbs in question: synonyms, co-hyponyms, hyper/hyponyms, antonyms, or none. GPT-4 achieved high correlations overall, but performance was considerably weaker for antonyms (see Fig. 3). Weaker performance for antonyms has been observed for other work on estimating similarity using distributional information (Dou et al., 2018). Specifically, GPT-4 tended to rate antonym pairs as more similar on average than humans did (mean difference = 2.53, SD = 1.64).

As with SimLex999, qualitative inspection revealed that a number of GPT-4’s ratings diverge not only for antonyms, but for other verb pairs that form semantic “complements” (e.g., “incline/decline”, “win/defeat”, “reap/sow”, “push/tug”, “die/kill”, “multiply/divide”, and “spring/fall”). In each of these cases, GPT-4 rated the pair as more similar than humans did.

*RAW-C*. GPT-4’s performance on the RAW-C dataset was higher (*rho* = 0.82) than both human inter-annotator agreement (*rho =* 0.79) and past language models tested (*rho* = 0.58). However, motivated by past work (Trott & Bergen, 2021; Trott & Bergen, 2023), I analyzed whether absolute errors were larger for contexts in which the meanings of the ambiguous word were distinct (e.g., “brain cell” vs. “prison cell’) than contexts in which the meanings were the same (e.g., “brain cell” vs. “skin cell”). Indeed, a linear regression demonstrated that errors were smaller on average for Same Sense contexts [*β =* – 0.53, SE = 0.04,* p* < .001].

I then asked whether human relatedness ratings varied significantly as a function of Same vs. Different Sense, *independent* of the effect of GPT-4’s relatedness ratings. Also consistent with past work (Trott & Bergen, 2023; Trott & Bergen, 2021), a linear regression predicting human relatedness suggested independent effects of each factor: GPT-4 relatedness [*β* = 0.95, SE = 0.05,* p* < .001] and Same Sense [*β* = 0.97, SE = 0.28, *p* < .001]. This suggests that GPT-4’s ratings fail to fully account for a psychological effect of whether two contexts convey the same or different meanings (Trott & Bergen, 2023); however, even within Same Sense pairs, GPT-4’s judgments significantly predicted human relatedness judgments [*β* = 0.34, SE = 0.06, *p* < .001].

Finally, a linear regression including an interaction between GPT-4 rating and Same Sense (along with main effects of each factor) revealed significant effects of each term: GPT-4 relatedness [*β* = 1.19, SE = 0.06,* p* < .001], Same Sense [*β =* 3.48, SE = 0.34, *p* < .001], and the interaction between GPT-4 relatedness and Same Sense [*β* = -0.85, SE = 0.11,* p* < .001]. The intercept is – 1.24, i.e., the estimated human relatedness for a *different* sense pair that received a rating of 0 from GPT-4 would be – 1.24. Put together, these effects can be interpreted as follows: for each 1-unit increase in GPT-4 relatedness ratings, human judgments of relatedness increase by approximately 1.19; additionally, holding GPT-4 judgments of relatedness constant, human judgments about the relatedness of Same Sense pairs are 3.48 higher on average; and finally, the interaction tempers this same-sense effect by a factor of – .85. More concretely: a GPT-4 rating of 3 for a *same sense* pair should yield a human relatedness judgment of approximately 3.3, while the same rating for a *different sense* pair should yield a human relatedness judgment of approximately 2.33.

This result is also consistent with qualitative inspection of the top 20 items with the highest error. In each case, GPT-4 systematically *overestimated* relatedness judgments for contexts conveying different meanings (e.g., “red *cape*” vs. “rocky *cape*”, or “*toast* the strudel” vs. “*toast* the host”).

#### Substitution analysis

Another way to evaluate the validity (and utility) of LLM-generated norms is to ask whether, and to what extent, they can be used as *substitutes* for human-generated norms in a statistical analysis. That is, if an analysis relied on LLM-generated norms instead of human-generated norms, how much would the results change? For example, a change in the *sign* of a coefficient estimate would be evidence that LLM-generated norms might lead to qualitatively different inferences; a small change in the *magnitude* of a coefficient estimate could be concerning, but perhaps less so than a change in its sign.

*Iconicity*. Winter et al. (2023) report the results of an analysis predicting human iconicity ratings as a function of multiple predictors: sensory experience (Juhasz & Yap, 2013) humor (Engelthaler & Hills, 2018), log letter frequency (Dingemanse & Thompson, 2020), concreteness (Brysbaert et al., 2014a, b), log word frequency (Brysbaert & New, 2009), average radius co-occurrence, or “ARC”^10^ (Shaoul & Westbury, 2010), age of acquisition (Kuperman et al., 2012), and part-of-speech. I replicated this analysis of human iconicity ratings using the data provided by the authors;^11^ as in the original article, all predictors were *z*-scored. Then, I conducted an identical analysis using LLM-generated iconicity as the target variable.

**Fig. 4:**
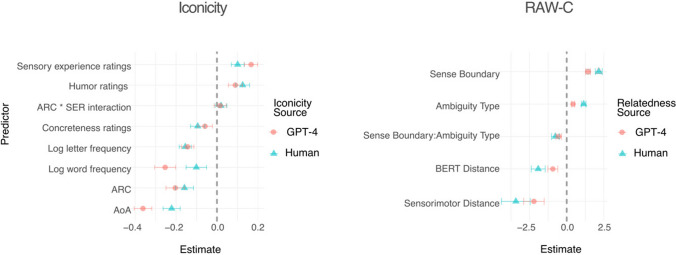
Coefficient values for statistical models predicting iconicity (*left*) and relatedness values (*right*). Iconicity and relatedness values were generated using either LLMs (*red circles*) or humans (*blue triangles*). *Error bars* represent two standard errors. As depicted, none of the coefficients switched their direction when LLM-generated norms were substituted for the dependent variable; however, select predictors did change magnitude depending on whether the dependent variable relied on LLM-generated or human-generated norms

The key question was whether coefficient estimates for a model predicting human-generated iconicity ratings would be different in sign or magnitude from those in a model predicting LLM-generated iconicity ratings. As depicted in Fig. 4, none of the coefficient estimates switched their *sign* (i.e., no predictors had negative coefficients for one measure of iconicity, and positive coefficients for the other measure). Following the authors’ convention, part-of-speech is not included in the figure.

Following past work (Clogg et al., 1995; Paternoster et al., 1998), differences in *magnitude* were assessed using a two-sided *z*-test:^12^

$$z= \frac{{\beta }_{1}- {\beta }_{2 }}{\sqrt{{SE}_{1}^{2}-S{E}_{2}^{2}}}$$

Using a standard significance threshold of *p* < .05, three predictors were found to have significantly difference coefficient estimates across models: log word frequency (*z* = 4.24, *p* < .001), age of acquisition (*z* = 4.67, *p* < .001), and sensory experience (*z* = – 2.93, *p* < .003). The remaining five predictors had coefficient estimates that were not significantly different (*p* > .1) across models. In other words, five of the predictors had stable coefficients regardless of whether they were used to predict human-generated iconicity ratings or LLM-generated iconicity ratings.

*Sensorimotor distance*. Following Wingfield & Connell (2022), Trott & Bergen (2022) used the contextualized sensorimotor norms to construct a measure of contextualized sensorimotor distance: the cosine distance between the 11-dimensional sensorimotor norms for each context in which an ambiguous word appeared. They demonstrated that this measure was predictive of relatedness judgments for those contexts (Trott & Bergen, 2021) above and beyond other measures, such as whether or not the contexts corresponded to the same sense, the kind of ambiguity expressed (homonymy vs. polysemy), and the cosine distance between BERT’s contextualized embeddings for those words.

To replicate this analysis, I first calculated contextualized sensorimotor distance using both the human-generated norms and the LLM-generated norms.^13^ These measures were relatively well-correlated (*rho* = 0.58, *p* < .001). I then built two different regression models predicting human judgments of contextual relatedness. Each model contained the following factors: Cosine Distance (measured by BERT), Sense Boundary (Same vs. Different Sense), Ambiguity Type (Homonymy vs. Polysemy), and an interaction between the latter two factors. The models differed in *which* measure of contextualized sensorimotor distance they used (i.e., relying on the human-generated vs. LLM-generated norms).

First, both models achieved comparable fits (*R*^*2*^_LLM_ *= 0.718, R*^*2*^_human_ *= 0.719*). The coefficient for contextualized sensorimotor distance was significantly negative for the model relying on LLM-generated norms [*β* = – 2.36, SE = 0.341, *p* < .001] and the model relying on human-generated norms [*β* = – 3.42, SE = 0.483, *p* < .001]. A *z*-test comparing these coefficient values was approaching significance (*z* = – 1.8, *p* = 0.07); this is consistent with a small but real difference in magnitudes between the estimates, but could also be consistent with sampling error.

*Contextual Relatedness.* I replicated the analysis above focusing on the contrast between LLM-generated and human-generated *relatedness*. Here, I constructed two linear regression models with identical predictors (BERT Distance, Sense Boundary, Ambiguity Type, an interaction between Sense Boundary and Ambiguity Type, and Sensorimotor Distance as measured by humans); the key difference was whether the dependent variable was LLM-generated relatedness or human-generated relatedness, i.e., the original RAW-C norms (Trott & Bergen, 2021).

As depicted in Fig. 4, none of the coefficients for the predictors changed sign across the models. However, a *z*-test did reveal significant changes in the magnitude of the coefficients for four of the five predictors: Sense Boundary (*z* = 5.03, *p* < .001), Ambiguity Type (*z* = 7.54, *p* < .001), BERT Distance (*z* = – 3.42, *p* < 0.001), and Sensorimotor Distance (*z* = – 2.05, *p* = .04). Notably, the effect of each predictor was *larger* when predicting human-generated relatedness; in the case of Sense Boundary and Ambiguity Type, this is consistent with past work (Trott & Bergen, 2023) suggesting that human semantic representations are influenced more by category boundaries (e.g., between distinct meanings of a word) than LLM representations.

**Fig. 5:**
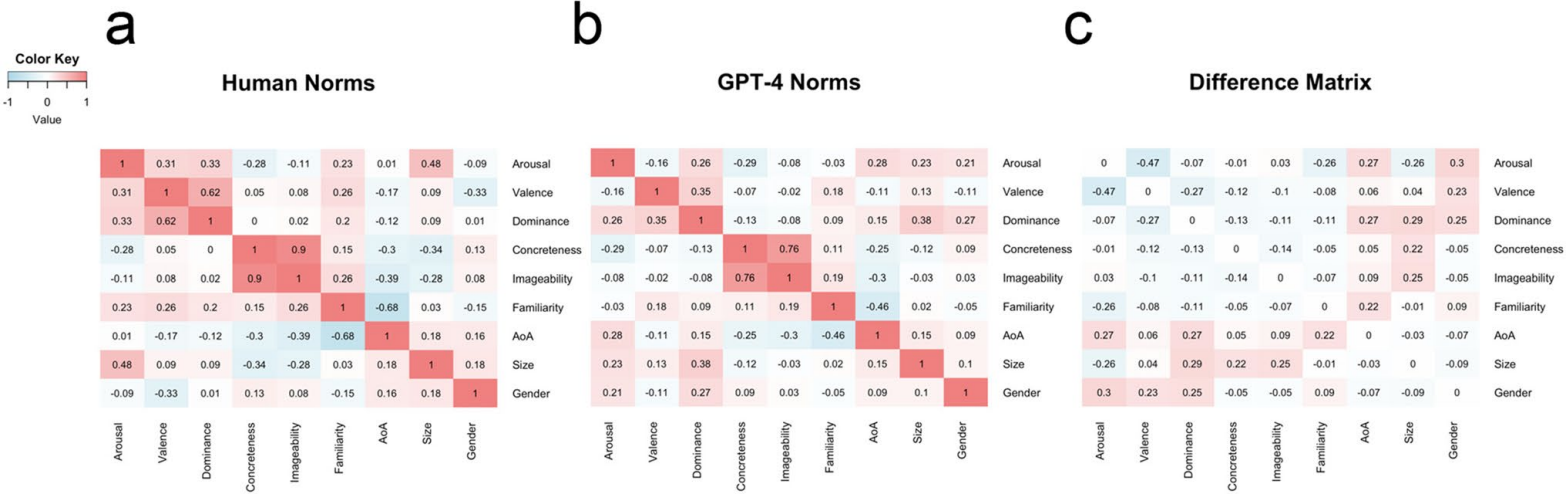
Correlation matrices for the nine Glasgow dimensions using human-generated norms (**a**) and LLM-generated norms (**b**). **c** The *difference* between these matrices (GPT-4 correlation – human correlation): a positive value means the dimensions were more positively correlated using GPT-4 norms, whereas a negative value means the dimensions were more positively correlated using human norms

*Glasgow Norms*. For the Glasgow Norms, I asked to what extent human-generated norms and LLM-generated norms reflected analogous semantic structure, i.e., whether the correlations *between* each of the nine dimensions (for human-generated norms) could be accurately reconstructed from the LLM-generated norms. The logic behind this approach was similar to representational similarity analysis, or “RSA” (Kriegeskorte et al., 2008). First, I constructed a correlation matrix between all nine dimensions using the human norms (see Fig. 5a). This reveals which psycholinguistic dimensions are positively correlated (e.g., imageability and concreteness) and which are negatively correlated (e.g., age of acquisition and familiarity), and to what degree. I then constructed an analogous matrix using the LLM-generated norms for each dimension (see Fig. 5b).

To test whether these matrices were more similar than one would expect by chance, I used a Mantel test of matrix similarity. A Mantel test calculates a correlation coefficient (e.g., Pearson’s *r*) between the off-diagonal cells across two matrices (the diagonals are excluded because they would artificially inflate the correlation value). This correlation coefficient is then compared to the distribution of correlation coefficients that result from randomly permuting one of the matrices and running the same procedure. Using 1000 random permutations, I found that the human correlation matrix was significantly correlated with the LLM-generated correlation matrix (*r* = 0.65,* p* < .001). That is, in addition to correlating with the original dimensions (see Fig. 2), the correlations *between* LLM-generated dimensions capture some of the structure of the original human dimensions.

That said, there were several notable cases in which GPT-4’s correlations departed substantially from human correlations. For example, arousal and valence were positively correlated in the human norms (*r =* 0.31), but (weakly) negatively correlated in the LLM-generated norms (*r* = – .16). In other cases, the coefficients had the same *sign* but varied in *magnitude*: for example, semantic size and semantic dominance were very weakly correlated in the human norms (*r* = 0.09, *p* < .01), whereas this correlation was somewhat stronger in the LLM-generated norms (*r* = 0.38, *p* < .001).

## General discussion

The primary question of the current work was whether LLMs could be used to *augment* the creation of large-scale psycholinguistic datasets, particularly those involving contextualized judgments. Focusing on six datasets (comprising 24 semantic dimensions total), I approached this question in the following way. First, in a series of pre-registered analyses, I found that LLM-generated norms were positively correlated with human judgments across all 24 dimensions of all datasets. Degree-of-fit ranged considerably across dimensions (see Table 1); however, where a baseline of human inter-annotator agreement was available, I found that LLM-generated norms approached—and in five cases *exceeded*—this baseline.

Second, for select datasets, I conducted exploratory analyses investigating where LLM-generated norms diverged from human judgments. For similarity judgments, divergences were largest for concrete words (SimLex999) and antonyms (SimVerb3500); both findings were consistent with past work (Utsumi, 2020; Dou et al., 2018). For contextualized relatedness judgments, divergences were most pronounced at *sense boundaries*, also consistent with past work (Trott & Bergen, 2023). Finally, I performed a novel *substitution analysis*, which asked whether LLM-generated norms could be substituted for human-generated norms as either an independent or dependent variable in a statistical modeling framework. In each of the substitutions performed, using LLM-generated norms did not result in changes of the direction (i.e., *sign*) for any coefficients across models (see Fig. 4). However, there were significant changes in the *magnitude* of coefficients for select predictors, such as age of acquisition and log word frequency (for predicting human-generated vs. LLM-generated iconicity norms).

### Are LLM-generated norms viable?

The question of viability depends on both theoretical and practical factors. First, how *successfully* can GPT-4 reproduce existing human judgments? Second, how *easy* is GPT-4 to use in this way, and how would that compare to collecting human judgments at scale? Third, how *expensive* is GPT-4 to use, and how does that compare to collecting human judgments at scale? I consider these factors in the sections below. Note that this discussion focuses on *written English stimuli*; other issues (e.g., external validity) are explored in the “Limitations” section.

#### Empirical success

Overall, these results are promising. GPT-4 achieved comparable (or superior) performance with human annotators in five of the six datasets where inter-annotator agreement was available. Additionally, the fact that LLM-generated norms could be substituted for human norms in a statistical model without changing the *sign* of any coefficients in the model suggests that these norms could be used to help drive theoretical inferences about how psycholinguistic variables relate to one another. Further, GPT-4 was presented with instructions that were identical (or nearly identical) to those presented to human participants. Thus, these results may reflect a *lower-bound* on GPT-4’s ability to produce aligned judgments. Better results could be obtained with alternative prompting methods (Reynolds & McDonell, 2021), well-chosen examples (Brown et al., 2020), or other ways of extracting LLM output (Hu & Levy, 2023).

Of course, the correlation with human norms was far from perfect, particularly for dimensions such as *semantic dominance* (though it is unclear what human inter-annotator agreement was for these dimensions). This raises an important question about the degree-of-fit required to augment datasets with LLM-generated norms: how successful must an LLM be—relative to a human baseline—to be used either in norming stimuli or increasing the size of a dataset? This question depends on a researcher’s goals and on the degree of precision required. Notably, LLM-generated norms performed much better for some dimensions than others. If this gradient in performance is systematic, then perhaps researchers could rely more on LLMs for those specific dimensions (e.g., contextualized concreteness), and focus their energies on collecting human data for other dimensions (e.g., semantic dominance); again, in each case, comparison to a human baseline would be essential.

Further, the *error analysis* suggests that LLMs perform better for some kinds of words (e.g., abstract words) than others (e.g., concrete words and antonyms); these findings are consistent with past work (Utsumi, 2020; Trott & Bergen, 2023), and, if they are replicable, suggest another path forward—perhaps LLM-generated norms could be relied on more for certain kinds of items or relations than others. Finally, substitution analyses could be performed to quantify the divergence in theoretical inferences one would obtain when relying on LLM-generated norms in place of human-generated norms.

#### Ease of use

One benefit of modern foundational LLMs is that users do not need to train their own model; in the case of GPT-4, users can access the model and produce output using either a web interface or a Python API. Intuitively, this seems easier to use than older models, though it is more challenging to compare to the ease of collecting judgments from human participants (e.g., over Amazon Mechanical Turk). Relying on a model like GPT-4 likely requires some Python programming knowledge, as well as basic familiarity with how LLMs work. On the other hand, collecting judgments online requires designing a survey interface (e.g., using Qualtrics) and addressing difficult issues like participant exclusion (Webb & Tangney, 2022). Ultimately, the question of which source is *easier* is ripe for empirical investigation. Researchers could combine qualitative and quantitative approaches to conduct a usability study and identify significant bottlenecks in each approach.

#### Cost

Running these analyses required access to the OpenAI API. According to OpenAI,^14^ GPT-4 costs $0.03/1000 for prompt tokens and $0.06/1000 for sampled tokens. Based on the number of tokens in the instructions and prompts for each dimension of each task,^15^ this results in a total prompt cost of $300.31. For token generation, I allowed GPT-4 to generate up to ten tokens for each judgment; at a rate of $0.06/1000 per generated token, this amounted to $19.63. Altogether, using GPT-4 to collect the total set of judgments cost approximately $319.94. (Of course, it is possible that the cost of GPT-4 will decrease in the future, or that freely available models will become more powerful; both possibilities would make LLM norms comparative cheaper, and thus this estimate should be considered a conservative, pessimistic one regarding LLM costs. See also Supplementary Analysis 2 for an analysis using a smaller, cheaper model.)

Estimating a cost for human-generated norms is more challenging, and relies on several assumptions. The minimal cost could be estimated assuming zero exclusions, a single judgment per word, a payment of federal minimum wage ($7.25 per hour), and relatively fast time per judgment (e.g., 5 s): at this rate, 32,000 judgments would take approximately 44.44 h, which would cost $329.19—about $10 more than the estimate for the LLM-generated norms.

However, this estimate is optimistic. First, a researcher would likely need to exclude at least some judgments. Recent work (Webb & Tangney, 2022) estimated an exclusion rate for Amazon Mechanical Turk as high as over 90%; exclusion rates for the original datasets considered here ranged from 11% (Winter et al., 2023) to 25% (Trott & Bergen, 2022). Second, the amount of time required to respond to an item will depend on the judgment in question; for single words, 5 s is reasonable (e.g., the average response time in Winter et al., 2023 was under 5 s), but a longer sentence or passage will naturally take longer to read and produce a judgment about.

Additionally, a single judgment per item is unusual. For example, Winter et al. (2023) and Trott & Bergen (2022) collected at least ten judgments per item. The number required will depend on the *relative precision* of any given human judgment. Recall that for a number of datasets, GPT-4’s correlation with the human mean was higher than the average inter-annotator agreement (see Fig. 1). Thus, a relevant question is how many human judgments are needed for a given judgment type to attain the same degree of reliability as LLM-generated judgments. Future work could address this question empirically: if this ratio is higher than 1 (i.e., a single human judgment is, on average, less reliable than an LLM-generated judgment), then more than a single human judgment would be required to attain comparable reliability, therefore raising the cost of human-collected data. If the ratio is lower than 1 (single human judgments are more reliable than LLM-generated judgments), then LLM-generated judgments would not necessarily be a useful or cost-effective contribution. This last question is also interesting from a theoretical perspective, as it connects to the notion of the “wisdom of the crowd” (Stroop, 1932). LLMs are trained on many more word tokens, from more language producers, than any given human observes; for certain tasks, then, it is possible that their output represents the average guess of multiple language producers (Dillion et al., 2023).

Ultimately, better characterization of the factors described here—empirical success, ease of use, and financial cost—would allow researchers to make informed cost/benefit analyses when determining how to create their normed stimuli. Additional questions about viability are explored in the section below.

### Limitations and future work

The work described here has various number of limitations, which also raise questions and interesting directions for future work.

#### Limited generalizability

One key limitation is that most large language models like GPT-4 are trained primarily on written English text produced by a relatively biased subset of English speakers (Bender et al., 2021; Chang & Bergen, 2023). Because of this, the output produced by most LLMs are limited to English; within English-speaking communities, they also under-represent the perspectives of traditionally marginalized groups (Groenwold et al., 2020). Further, because LLMs are trained on written text, they fail to capture important variation in spoken language, and cannot be used to model judgments about signed languages at all (Vinson et al., 2008). In addition to concerns about perpetuating bias or producing toxic speech (Bender et al., 2021), this raises a concern about the *external validity* of LLM-generated samples.

Of course, concerns about external validity are not unique to LLMs. Experimental samples in psychology and cognitive science have traditionally over-represented so-called “WEIRD” (Western, Educated, Industrialized, Rich, and Democratic) populations (Henrich et al., 2010). Additionally, English specifically has often been treated as the “default” language of study (Bender, 2009; Anand et al., 2020; Blasi et al., 2022). In terms of English psycholinguistic norms in particular—the subject of this paper—it is unclear to what extent the samples used to generate these norms are representative of the broader English-speaking population.

Altogether, this suggests that researchers should apply exercise caution when making claims about the generalizability of findings obtained on LLM-generated samples—just as they should for samples obtained from WEIRD populations.

#### Limited understanding

LLMs lack both embodied and interactional experience, leading many to question whether they exhibit true “understanding” of human language (Bender & Koller, 2020; Mollo & Millière, 2023; Mitchell & Krakauer, 2023). Additionally, LLMs may lack “common sense” knowledge (Forbes et al., 2019), suggesting that certain aspects of human knowledge and reasoning cannot be learned from linguistic input alone. However, others have argued that LLMs do acquire relevant aspects of linguistic meaning (Piantadosi & Hill, 2022) and even reasoning ability (Manning, 2022). Empirically, evidence is mixed: LLMs do perform surprisingly well on select tasks requiring linguistic or even social reasoning (Hu et al., 2022; Trott et al., 2022), though typically under-perform human benchmarks (Jones et al., 2022). As Pavlick (2023) notes, this debate is far from resolved. Ultimately, a resolution will hinge not only on a priori arguments about what could in principle be learned from language, but empirical investigations into both how LLMs behave and which representations guide that behavior.

In terms of the current work, one central question is whether and what LLMs understand about the words and constructs for which they are producing norms. Empirically, the results presented here demonstrate that LLMs perform well overall, and further, that performance is better for some constructs (e.g., concreteness) than others (e.g., dominance), and that their judgments are also dependent on the types of words or relations in question (e.g., LLMs perform better for synonyms than antonyms). One interpretation of these results is that LLMs thus “understand” these words and constructs moderately well, but better in some cases than others. However, because “understanding” remains a contested concept, consensus on that interpretation may be unlikely. As Pavlick (2023) notes, addressing this question is likely to require considerable empirical investigation—and also, crucially, a more complete theory of exactly what and how *humans* understand language. This issue is explored at greater length in the section below entitled “Which types of judgments can LLMs make?”

#### Which types of judgments can LLMs make?

As noted in the Introduction, there is growing interest in using LLMs to make a variety of judgments about written stimuli (Törnberg, 2023, Zhu et al., 2023; Gilardi et al., 2023), including judgments about the morality of different situations (Dillion et al., 2023). Should LLMs be relied upon more for certain kinds of judgments than others?

It seems uncontroversial that LLMs could produce reliable judgments about an English word’s part-of-speech; it is less clear whether LLMs can (or should) be relied upon for judgments about the ethics or morality of a written scenario. This question also connects to the issue of representativeness: according to recent work, LLM-generated moral evaluations correlate well with norms generated by English-speaking participants (Dillion et al., 2023), but less well with judgments produced by speakers around the world (Ramezani & Xu, 2023); this is not surprising, given that moral judgments vary considerably by culture (Henrich et al., 2010; Awad et al., 2020). Thus, the issue appears to encapsulate both *construct validity* (whether LLM-generated norms are valid operationalizations of the underlying theoretical construct) and *external validity* (whether those norms reflect the population of interest). Because external validity has already been discussed above, I focus here on construct validity.

There are at least two approaches to answering this question, which relate to different dimensions of construct validity. One is empirical and atheoretical: LLMs can be relied upon to the extent that their judgments correlate with human judgments. This echoes the “duck test” position described in other work (Trott et al., 2022), i.e., if an LLM produces judgments that correspond to human-generated judgments, then the LLM is a reliable source of those judgments. Depending on the empirical analysis in question, this could be analogized to establishing the *reliability* of a measure (e.g., inter-annotator agreement; see Fig. 1) or establishing the *predictive validity* of a measure (e.g., its ability to predict outcomes of interest; see Fig. 4 and the corresponding substitution analysis). This empirical approach has the advantage of offering a specific, measurable criterion for determining whether or not LLM-generated norms are suitable. However, a disadvantage is that it does not contend with theoretical objections whether an LLM is in principle capable of providing certain kinds of judgments.

The other approach is conceptual and focuses on questions of a priori validity: given the limitations of their training data (e.g., solely linguistic input), then LLMs can perhaps be relied upon for judgments about *language*, but not judgments about *the world* or *human society*. This objection could be analogized to the question of *face validity*: there are certain theoretical constructs for which LLM-generated judgments simply seem implausible or inherently unreliable (e.g., perhaps moral norms). Other constructs, like iconicity, are on the margin: judgments about iconicity require knowledge both about a word’s meaning (which may in part be inferable from distributional statistics) and its form (which is not explicitly encoded in an LLM); at the same time, there is some evidence that LLMs do acquire knowledge about the spelling of their tokens (Kaushal & Mahowald, 2022), which could form the foundation of knowledge about iconicity. Overall, this a priori approach would advocate for using LLM-generated norms only when LLMs could be considered a plausible, reliable source of knowledge about a domain—independent of their empirical performance. This approach has the advantage of engaging with issues of theoretical plausibility, but is disadvantaged by the fact that it is not always clear how to establish and agree upon clear criteria for something like face validity.

Of course, it is possible the correct approach lies somewhere in the middle. As described in related work (Trott et al., 2022), the question of whether or not LLM-generated judgments exhibit construct validity could be addressed by comparing humans and LLMs at multiple levels of analysis. Drawing on Marr’s levels of analysis (Marr & Poggio, 1976), one might differentiate between analogous *input/output behaviors* (the “computational” level) and analogous *representational* mechanisms underlying that behavior (the “representational” or “algorithmic” level). The current work focuses on the computational level of analysis, quantifying the empirical correlation between human-generated and LLM-generated judgments. Future work could aim to characterize the representational analogies or disanalogies, using empirical and theoretical perspectives (Mahowald et al., 2023; Pavlick, 2023).

#### Data leakage

Models like GPT-4 are proprietary, both in terms of their trained parameter values and the details of their training data. This makes *data leakage* a cause for concern, i.e., overlap between the training set and the test set. Data leakage can lead to *overestimates* of an LLM’s abilities—for example, if GPT-4 was trained on the RAW-C dataset, it would not be surprising that it could regenerate human norms with high accuracy. In the current work, I used at least one dataset that was released *after* GPT-4 was trained (Winter et al., 2023), which suggests that data leakage could not be a concern for the iconicity norms specifically. Further, as illustrated in Supplementary Analysis 1, the fit between GPT-4’s iconicity ratings and human iconicity ratings cannot be explained by iconicity correlates or by the presence of words in pre-existing iconicity datasets. Additionally, Supplementary Analysis 3 provides further evidence against the possibility of data contamination, using a recently pioneered detection method (Golchin & Surdeanu, 2023). However, future work should aim to address this issue by continuing to evaluate an LLM’s performance on norms that are unlikely to have been observed in its training set.

#### Choice of model

The current work relied on GPT-4, a state-of-the-art LLM released by OpenAI. As noted in the Methods section, one limitation of GPT-4 (along with other OpenAI models) is that the details of its architecture or training data have not been made public. Further, after pre-training, GPT-4 was trained using “reinforcement learning with human feedback (RLHF), in which model weights are iteratively updated according to explicit human feedback about which model outputs are appropriate or inappropriate; the details of this feedback are also not entirely open. Lack of model transparency could be a concern for many scientific questions about LLM performance. For example, if one is interested in how exposure specific kinds (or amounts) of linguistic input facilitates performance on a task, then not knowing exactly what a model is trained on impedes one’s ability to make relevant scientific inferences. Similarly, a process like RLHF introduces features other than pure distributional statistics into the training signal; thus, if one’s question concerns the sufficiency of more classical models of learning from statistical distributions alone, then a model trained with RLHF is likely not suitable.

The primary research question of the current work was whether and to what extent a state-of-the-art LLM could reproduce human psycholinguistic judgments. Answering this question does not hinge critically on a model’s architecture or training regime; the input data does matter, but only insofar as data contamination is a concern (see Supplementary Analysis 1). In contrast, the question does hinge on operationalizing “state-of-the-art” and “LLM”; I selected GPT-4, which has achieved strong performance on a number of benchmarks and real-world tasks, and which is considered an LLM. Future work would benefit from comparison to other models, including smaller GPT models (e.g., GPT-3) as well as open-source LLMs.

Finally, future work interested in the issue of which representations mediate input/output behavior observed here may find it useful to make use of open-source models with accessible internal states. The current work relied on error analyses to make inferences about the processes giving rise to behavior; this is analogous to a dominant approach taken in cognitive psychology, in which internal states cannot be directly observed and must be inferred from behavior. In the case of LLMs, these questions could also be addressed by analyzing internal states directly (perhaps more analogous to neuro-imaging approaches in human psychology) and even intervened upon (i.e., as in optogenetics); this last methodology would be most effective at establishing *causal mechanisms* underlying certain behaviors, and is also an approach that is usually unethical to implement in humans.

#### Towards a theory of prompting

Relatedly, it is important to note that the current work prompted GPT-4 with the same (or only slightly modified, in some cases) instructions given to human participants. This was done to establish the initial viability of LLM-generated norms and to avoid the possibility of introducing either type I or type II errors by manipulating the prompt. Given that it is not entirely clear which instructional changes would bias LLMs in which direction, this was taken as a “neutral” starting point for establishing a research program focusing on interrogating the reliability of LLM-generated norms.

However, there is some evidence that alternative prompting approaches (Hu & Levy, 2023; Reynolds & McDonell, 2021) lead to more accurate results; further, prompts with embedded, well-chosen exemplars (e.g., “few-shot”) may improve LLM performance (Brown et al., 2020). It is possible that LLMs may generate more reliable norms using *different* instructions than those given to human participants and that the current work is in a “local optimum” in terms of prompting. Alternatively, other “adversarial” prompts could impair LLM performance, i.e., lead the LLM to produce norms that are decorrelated or negatively correlated with human norms. Relatedly, alternative “temperature” settings could be used: rather than selecting the most probable token in a given context (a temperature of 0), the model could be allowed to generate multiple tokens at a higher temperature; this could give a better indication of the underlying probability distribution and perhaps yield more accurate judgments, e.g., in the cases when the most likely token is only slightly more probable than the second most likely token. One open question is whether variance in GPT-4 judgments under higher temperature settings is correlated with variance in human judgments for a given item. Future work should explore the parameterization space more thoroughly, ideally with the ultimate aim of identifying a generalizable theory of prompting. The methods developed and presented in this paper could be used as a framework for evaluating the success of each approach.

#### Augmentation vs. replacement

Some recent papers have asked whether LLMs could be used to replace humans, both as experimental participants and within the labor force (Eloundou et al., 2023). Throughout this manuscript, however, I have approached this as a question as *augmentation*. Because of the limitations described above—questions of external validity, precision, etc.—it seems premature to seek to *replace* human participants entirely. Instead, as Dillion et al. (2023) notes, perhaps LLMs could be strategically deployed at select stages of the research cycle (e.g., pilot studies), or used in concert with human participants to reduce the cost of norming stimuli. For example, if LLM-generated judgments are sufficiently reliable, then rather than collecting ten human judgments per word, researchers could collect five human judgments and combine these with LLM-generated judgments. This would decrease the costs of data collection and allow researchers to allocate expenses towards other stages of the research cycle.

Crucially, however, an estimate of the reliability of LLM-generated norms depends upon a human “gold standard” with which to evaluate those norms—which is a key argument for keeping “humans in the loop”. Without such a corrective baseline, our datasets may “drift” towards the statistical biases of LLMs (see Error analysis), terraforming the conceptual landscape of our scientific theories. This also reinforces the importance of ensuring the reliability and generalizability of our *human* samples (Henrich et al., 2010), as well as accounting for individual variability or “inter-annotator *disagreement*” in lexical representations: if individual humans (within or across populations) cannot agree on a judgment, then what, exactly, is in a norm?

## Conclusion

Psycholinguists rely on human judgments of lexical properties to help norm their experimental stimuli and conduct large-scale statistical analyses of the lexicon (Xu et al., 2020; Winter et al., 2023). However, these datasets are challenging and time-consuming to construct, particularly for *contextualized* judgments. One solution is to augment contextualized datasets with judgments generated by large language models (LLMs). I empirically investigated the viability of this solution for English datasets; the results suggest that in many cases, LLM-generated norms rival the reliability of norms generated by individual humans, and can even be substituted for human norms in statistical models without changing theoretical inferences. However, LLM-generated norms also diverge from human judgments in predictable ways, introducing statistical biases into their judgments. Moving forward, the Psycholinguistics community could benefit from more systematic investigation of the strengths and limitations of this approach, ideally keeping humans “in the loop” to avoid systematic drift of our datasets.

### Supplementary Information

Below is the link to the electronic supplementary material.

Supplementary file1 (DOCX 34 KB)

## Data Availability

The output of the GPT-4 norming process can be found on GitHub: https://github.com/seantrott/llm_norms.

## Footnotes

1 Assuming, of course, that the researcher is not primarily interested in the effect of these properties on the dependent variable of interest.

2 https://www.mturk.com/

3 Note that this is not an endorsement of paying a very low wage; this example is used simply to estimate a “lower-bound” on the amount a researcher would need to pay.

4 This description applies to *auto-regressive* LLMs specifically, which are trained to predict the *next* word. Some LLMs, such as BERT (Devlin et al., 2018), are “bidirectional” in that they can use information that comes before and after a “masked” word to predict the identity of the word (e.g., “The [MASK] sat on the mat”).

5 See the technical report: https://arxiv.org/abs/2303.08774.

6 https://openai.com/blog/openai-api

7 Note that this modification was simply to make it clear to the model that a number was expected; in the case of a human experiment, this would not have been necessary, as the sliding scale would have been visible to human participants.

8 Both changes were implemented to make the LLM’s goal more straightforward. In the first case, the prompt specified that a number (as opposed to a verbal description, e.g., “very concrete”) was required. In the case of Age of Acquisition, the original scale included ranges that I predicted might be unintuitive (e.g., a “7” referred to any age above 13), so this was the pre-registered prompt I decided on.

9 Leave-one-out is more comparable in that GPT-4 can be construed as a single “participant”, whose ratings we are comparing to the mean ratings of all other participants. It is also a more “generous” measure to human participants in that it tends to lead to a higher estimate than the average pairwise correlation between human participants. The question is thus where the quality of GPT-4’s data falls in the distribution of human participants.

10 ARC is a measure of semantic neighborhood density.

11 https://osf.io/qvw6u/

12 The primary reason for running a *z*-test here, rather than a *t* test, was based on the precedent from past work (Clogg et al., 1995). In practice, the key difference is which sampling distribution the resulting test statistic is compared to, i.e., either the standard normal distribution (in the case of a *z*-test) or a *t*-distribution with the appropriate degrees of freedom (in the case of a *t* test). Given that *t*-distributions have heavier tails, using a *t* test would generally work against finding significant differences in magnitude between models fit using the LLM-generated vs. human-generated norms. *P* values were calculated again using a *t* test (where degrees of freedom were estimated as the difference between the number of observations and the number of coefficients in the model minus one), and did not differ qualitatively from the results obtained using a standard normal distribution.

13 As in the original paper, this measure of contextualized sensorimotor distance included all 11 dimensions (i.e., all the perception norms and all the action norms).

14 https://help.openai.com/en/articles/7127956-how-much-does-gpt-4-cost

15 The number of tokens was estimated using *tiktoken*, a Python library (https://github.com/openai/tiktoken).

## Code availability

Code to reproduce the original GPT-4 norms (using the OpenAI API), along with the analyses described and visualizations shown in the paper, can also be found on GitHub: https://github.com/seantrott/llm_norms. The prompting method (and primary analyses) were pre-registered for: the iconicity norms (https://osf.io/wn9pv), SimVerb3500 (https://osf.io/dtekj), the contextualized sensorimotor norms (https://osf.io/2e3vk), and the Glasgow Norms (https://osf.io/3jvg6).
