# Supplementary material for: Can large language models help augment English psycholinguistic datasets?
Source: Behav Res Methods. 2024 Jan 23;56(6):6082–100. doi: 10.3758/s13428-024-02337-z (PMC11335796; doi:10.3758/s13428-024-02337-z)
Supplement: Supplementary file 1 — Supplementary file1 (DOCX 34 KB) [file 13428_2024_2337_MOESM1_ESM.docx]

**Supplementary Analysis 1**

In the primary manuscript, I found that GPT-4’s iconicity ratings were significant correlated with human iconicity ratings. One question is *how* GPT-4 is able to predict iconicity, given that GPT-4 does not have explicit phonological knowledge (or physical world experience). In this analysis, I attempt to rule out two potential *superficial* explanations for this correlation: data contamination (i.e., previously published iconicity datasets) and potential confounds (i.e., predicting a *correlate* of iconicity that can be estimated from distributional statistics).

**Analysis 1: Data Contamination**

One concern is that GPT-4 has simply “memorized” iconicity ratings for previously published datasets. Although Winter et al. (2023) published their ratings after GPT-4 was trained, it is possible that GPT-4 has memorized ratings from past datasets, e.g., Perlman et al. (2015). The most straightforward version of this claim would predict that: 1) GPT-4 has lower error in predicting ratings from previously published datasets than the new ratings (Winter et al. (2023); and 2) GPT-4 is essentially at chance when predicting new ratings in Winter et al. (2023).

To test the first claim, I first identified which words appeared in Perlman et al. (2015); I call this variable Already Published. Then, I calculated the *absolute error* between GPT-4’s iconicity ratings and human iconicity ratings for Winter et al. (2023). A linear regression predicting Absolute Error from Already Published found no significant effect of a word’s ratings already having been published in Perlman et al. (2015) (*p < .001*).

To test the second claim, I removed words from Winter et al. (2023) that also appeared in Perlman et al. (2015). I then recalculated Spearman’s *rho* between GPT-4 iconicity ratings and human iconicity ratings; *rho* was significantly positive (*rho = 0.59, p < .001*).

**Analysis 2: Correlates of iconicity**

Another potential concern is that GPT-4 is predicting some *correlate* of iconicity, but not iconicity itself. For example, perhaps the limited examples in the instructions provided to GPT-4 (and human participants) prompted GPT-4 to associate “high iconicity” and “low iconicity” words with some other correlate of iconicity that may be more directly accessible to GPT-4, such as frequency or concreteness. If this is true, then the relationship between human judgments and GPT-4 judgments in a regression model should no longer be significant once other known correlates of iconicity are accounted for.

To test this, I replicated the substitution analysis described in the primary manuscript, with one key difference: in addition to predicting GPT-4 iconicity ratings from a range of predictors (ARC, Humor, SER, Concreteness, Age of Acquisition, Log Word Frequency, Log Letter Frequency, and an ARC:SER interaction), I added *human* iconicity ratings as a predictor. Despite accounting for these other covariates, the model assigned a significantly positive coefficient to Human Rating [*β = 0.6, SE = 0.02, p < .001*].

**Discussion**

The goal of this Supplementary Analysis was to rule out two potential confounding explanations for *why* GPT-4 was able to predict human iconicity ratings. Although these analyses cannot entirely rule out the possibility of data contamination or a confound with iconicity, they do suggest that these explanations are less likely. GPT-4 ratings of iconicity were still predictive of human ratings even after removing previously published iconicity ratings from the dataset; additionally, there was a robust relationship between human and GPT-4 ratings even after accounting for other correlates of iconicity.

**Supplementary Analysis 2**

One question that arises is whether and to what extent the results presented here would generalize across different models. Large Language Models (LLMs) span a range of sizes, training regimes, and datasets. Typically, larger models perform better than smaller ones (Kaplan et al., 2020), though there are some exceptions to this rule (Kuribayashi et al., 2021). GPT-4 is one of the largest LLMs available, and thus it is possible that GPT-4’s performance represents the current “peak” of performance expected from current models without further fine-tuning or specialization.

In this analysis, I asked to what extent a cheaper, smaller model (GPT 3.5) could reproduce the performance displayed by GPT-4 in the primary manuscript. As a test case, I used GPT-3.5 “turbo” to produce relatedness ratings of ambiguous words from the RAW-C dataset, with the same prompting procedure used for GPT-4.

**Results**

Relatedness judgments produced by GPT 3.5 were well-correlated with human average relatedness judgments, using either Pearson’s *r* (*r*  = 0.83, p < .001) or Spearman’s *rho* (*rho*  = 0.83, p < .001). Human inter-annotator agreement for the RAW-C dataset was 0.79. Interestingly, GPT 3.5’s ratings were slightly more correlated with the human average than GPT-4’s ratings (approximately 0.82).

I then asked whether errors in these relatedness judgments exhibited a similar pattern as GPT-4’s relatedness judgments. As with GPT-4, absolute errors (i.e., the absolute difference from human relatedness judgments for a given sentence pair) were smaller on average for Same Sense contexts [*β = -0.48, SE = 0.05, p < .001*] than Different Sense contexts. Further, although GPT-3.5 relatedness judgments explained significant variance in human relatedness judgments, a linear regression suggested independent effects of Same Sense [*β = 0.89, SE = 0.08, p < .001*]. That is, like GPT-4, GPT-3.5 fails to fully account for a psychological effect of discrete sense boundaries (Trott & Bergen, 2023). Finally, within Same Sense pairs, GPT-3.5’s judgments significantly predicted human relatedness judgments [*β = 0.53, SE = 0.08, p < .001*]. This suggests that human relatedness judgments are not driven purely by a discrete variable representing whether two meanings are the same or not; even when two meanings are the *same*, variance in perceptions of relatedness is correlated with variance in GPT-3.5 relatedness judgments.

**Supplementary Analysis 3**

As noted in *Supplementary Analysis 1*, one concern that arises for select datasets is data contamination (or “data leakage”). If GPT-4 was trained on a dataset it is now being tested on, it is unsurprising (and uninteresting) that it is able to reproduce the norms with high accuracy. Data contamination can be ruled out for some datasets (e.g., iconicity) on the basis of when those datasets were released, as well as the fact that GPT-4 does not perform better for words with previously published iconicity ratings (see *Supplementary Analysis 1*). This shows that in principle, GPT-4 can perform well in the absence of data contamination. However, other datasets, such as the Glasgow Norms, could still have made it into GPT-4’s training data.

Unfortunately, identifying data contamination can be challenging. In this supplementary analysis, I adapt an approach pioneered by Golchin & Surdeanu (2023), in which an LLM is prompted to reproduce the exact sentences from a Natural Language Processing (NLP) task. The method works by referencing the specific dataset being reproduced (E.g., “the WNLI dataset”), and instructing the LLM to continue the prompt with the sentence from the dataset. The LLM’s continuation is then compared to the true continuation from the original dataset, e.g., using measures of string overlap (ROUGE-L). This metric of overlap is compared to the metric one would obtain if using the continuation generated using general instructions, i.e., without a “guided bias” towards data contamination. The logic is that if overlap improves when the dataset is biased towards exact reproduction, there is evidence of contamination. This approach is quite successful at detecting contamination (with success rates between 78% and 100%).

In the case of psycholinguistic norms—which are typically represented as *numbers* in a comma-separated values file rather than sentences in a text file—this approach must be adapted slightly. I used two different metrics of overlap to minimize the chance of a false negative (i.e., that true data contamination would not be detected). As in the original paper (Golchin & Surdeanu, 2023), each metric of data contamination was compared to the norms produced under general instruction.

**Methods**

I attempted to elicit direct reconstructions of the *.csv* file that GPT-4 would have been trained on, had it been exposed to a given dataset. I applied this method to the Glasgow Norms (Scott et al., 2019) using the following prompt structure:

"These are psycholinguistic norms from the Glasgow Norms dataset. Please continue each row with the correct number from the dataset.”^[[1]](#footnote-1)^

“word,Length,Arousal.M”

“{word},{length},"

The first line of the prompt makes reference to the dataset (Glasgow Norms) and asks GPT-4 to continue the row with the correct number from the dataset. The second line of the prompt lists the structure of the *.csv* file in question with the appropriate column headers. Note that this method was applied only to the arousal dimension, as this was the third column listed in the original .csv file. Thus, each other column all depend on having available values for the columns to their left; if these values were included (e.g., using the values from the original dataset), it could improve the model’s ability to reconstruct the values for reasons other than data contamination—namely, the model might have implicitly learned a correlation between specific dimensions (e.g., concreteness and age of acquisition) and use that correlation to predict or “fill in” numbers for the column in question.

Note that in several cases, GPT-4 did not generate a number in its response (e.g., “I’m sorry, I cannot answer this question”), resulting in a total of 859 responses.

**Results.**

In the original approach, Golchin & Surdeanu (2023) assess data contamination by measuring the degree of character overlap between the sentence in the original dataset and the LLM-generated sentence. It is challenging to apply this approach directly to comparing two different sources of numbers (i.e., the original norms and the LLM-generated completions). Here, I adopted two different assessment strategies.

**Analysis 1: Correlation Comparison.** First, I calculated the degree of correlation between GPT-4’s generated completions and the original human norms. Note that this is an imperfect measure of data contamination because a high correlation could be achieved simply by the model understanding what it is being asked to do, i.e., generate “arousal” values—which would be a signal that the model understands arousal and how to rate it on a scale (as in the primary manuscript). In this case, the degree of correlation was moderately high (*r*  = 0.53, p < .001), though crucially, *lower* than the correlation reported in the primary manuscript (*rho*  = 0.66, p < .001). That is, GPT-4’s degree of success in producing arousal norms was higher when asked to do so using general instructions then when biased towards exact reproduction of the dataset. As Golchin & Surdeanu (2023) argue, this suggests that the success of the original norms cannot be attributed directly to data contamination.

**Analysis 2: ROUGE-L Comparison.** Second, I calculated the degree of overlap between the string representing the original arousal norms (e.g., “2.4”) and the LLM-generated string (e.g., “2.5”) using the ROUGE-L score, as in Golchin & Surdeanu (2023). ROUGE-L measures the length of the largest common subsequence in both strings. Although ROUGE-L has a number of conceptual limitations for assessing tasks like paraphrase quality or translation fidelity, it is actually suitable to this purpose here, as it helps quantify the extent to which the LLM-generated norms are exact reproductions of the original norms. Importantly, this score was calculated for both the norms produced under general instruction (i.e., those reported in the original manuscript) and the norms produced using this “guided instruction” approach.

The mean ROUGE-L score was higher on average using the general instruction approach (M = 0.19, SD = 0.24) than the “guided instruction” approach (M = 0.23, SD = 0.26). This difference was significant using an independent samples t-test [*t(1716) = 3.41, p < .001*]. Again, this indicates that the “guided instruction” method (which was intentionally designed to elicit exact reproductions) did not result in closer reconstructions than the “general instruction” method used in the primary manuscript, which provides evidence against data contamination. More precisely, it is less likely that the *success* of the “general instruction” approach was due to GPT-4 having memorized the original dataset.

1. Note that new-lines were implemented using the newline (“\n”) character. [↑](#footnote-ref-1)
